# Discrimination and Nitric Oxide Inhibitory Activity Correlation of Ajwa Dates from Different Grades and Origin

**DOI:** 10.3390/molecules21111423

**Published:** 2016-10-28

**Authors:** Nur Ashikin Abdul-Hamid, Ahmed Mediani, M. Maulidiani, Faridah Abas, Intan Safinar Ismail, Khozirah Shaari, Nordin H. Lajis

**Affiliations:** 1 Laboratory of Natural Products, Institute of Bioscience, Universiti Putra Malaysia, 43400 Serdang, Malaysia; shikinhamid89@yahoo.com (N.A.A.H.); maulidiani@upm.edu.my (M.M.); safinar@upm.edu.my (I.S.I.); khozirah@yahoo.com.my (K.S.); 2 Department of Food Science, Faculty of Food Science and Technology, Universiti Putra Malaysia, 43400 Serdang, Malaysia; mediani82@yahoo.fr; 3 Department of Chemistry, Faculty of Science, Universiti Putra Malaysia, 43400 Serdang, Malaysia

**Keywords:** *Phoenix dactylifera*, Ajwa dates, RAW 264.7 cells, NMR metabolomics, nitric oxide

## Abstract

This study was aimed at examining the variations in the metabolite constituents of the different Ajwa grades and farm origins. It is also targeted at establishing the correlations between the metabolite contents and the grades and further to the nitric oxide (NO) inhibitory activity. Identification of the metabolites was generated using ^1^H-NMR spectroscopy metabolomics analyses utilizing multivariate methods. The NO inhibitory activity was determined using a Griess assay. Multivariate data analysis, for both supervised and unsupervised approaches, showed clusters among different grades of Ajwa dates obtained from different farms. The compounds that contribute towards the observed separation between Ajwa samples were suggested to be phenolic compounds, ascorbic acid and phenylalanine. Ajwa dates were shown to have different metabolite compositions and exhibited a wide range of NO inhibitory activity. It is also revealed that Ajwa Grade 1 from the al-Aliah farm exhibited more than 90% NO inhibitory activity compared to the other grades and origins. Phenolic compounds were among the compounds that played a role towards the greater capacity of NO inhibitory activity shown by Ajwa Grade 1 from the al-Aliah farm.

## 1. Introduction

Date palm (*Phoenix dactylifera* L.) is an important crop in the Middle East and many parts of the world. The fruits of this palm are the major source of income and food for the oasis farmers [1]. They consist of a seed covered with a fleshy pericarp, which makes up 85% to 90% of the date fruit weight. Traditionally, the extracts of the dates have been given to women after childbirth to stimulate the immune system [2]. Previous studies revealed that date palm fruits possess numerous beneficial properties, including anti-microbial and antimutagenic properties. In addition, dates have been shown to have important therapeutic values for glycaemia and lipid control in diabetic patients [3]. Their physical properties are useful indicators of the fruit maturity, degree of ripeness and for a prediction of shelf life. The chemical composition is an important criteria for nutritional significance and health benefits. Consideration of these criteria is essential for the sorting, grading and management of products for quality control. To achieve these, reliable approaches are necessary for the benefit of all relevant parties.

Metabolomics has been increasingly used to gain insight into the biochemical composition of crop plants. It has been used to determine and identify variations or similarities between different sample pools in a general manner, based on their metabolite finger prints [4]. In recent years, ^1^H-NMR spectroscopy has proven to provide reliable data for plant sample classification and subsequent identification of discriminating features. The use of ^1^H-NMR spectroscopy in metabolomics analysis offers several advantages over other techniques, owing to its simple sample preparation, large information content in single spectra and the ability to identify discriminating constituents from spectral features or covariance matrix analysis. Furthermore, ^1^H-NMR spectroscopic data are highly reproducible, thus allowing spectral comparison with those in the literature or databases [5]. It is also useful for correlating the chemical constituents of extracts with the biological activity.

The fruits of the Ajwa variety, which are specifically produced from the farms surrounding the city of Madinah al-Munawarah, Saudi Arabia, are considered to be special among the Muslim communities for the belief that they were the preferred fruits of the Prophet. They are highly acclaimed for their excellent health and medicinal attributes. Consequently, this particular variety has become the most sought after among the date fruits [6]. Ajwa dates are classified into different grades according to their size and external features, including moistness, the amount of flesh, color, uniformity of size and the absence of defects, and are priced at different ranges. Ajwa dates with a better quality belong to a better grade, hence being more expensive in the market. Unfortunately, there has not been any scientific method established for the grading practice other than by basing it on their external observable features. The Grade 1 dates are generally larger than those of Grades 2 and 3. They are moister, display less or no defects and are visibly more attractive than the lesser grades. In terms of their colors, the superior Grade 1 appeared darker than the other two, with Grade 3 being generally the lightest. The Grade 3 dates may be firm, but are pliable and may possess semi-dry calyx ends. The special position of Ajwa dates and the lack of scientific basis for its classification encouraged us to investigate this phenomenon, while at the same time attempting to identify the important markers for the different grades and correlate them with the qualities.

The most popular claim that consumption of Ajwa dates could prevent one from illnesses may be associated with the improvement of the immune function of the body against the invasion of foreign agents. Inflammation is one of the defense systems against stress inflicted by such invasion. Anti-inflammatory property can therefore become one of the factors that can be associated with protection from potential illnesses. It is believed that management of the excessive production of nitric oxide (NO) by macrophages should significantly aid in the treatment of inflammatory illnesses [7,8]. On the basis of these, the anti-inflammatory potential of Ajwa dates was evaluated in this study by measuring the NO inhibitory effect utilizing murine macrophage RAW 264.7 cells. The ^1^H-NMR-based metabolomics approach was used to discriminate different grades of Ajwa dates collected from different cultivation farms. In addition, the correlation between NO inhibitory activity and the metabolite profiles of the extracts was also examined.

## 2. Results and Discussion

### 2.1. Spectral Features and Assignment of Metabolites

**Figure 1:**
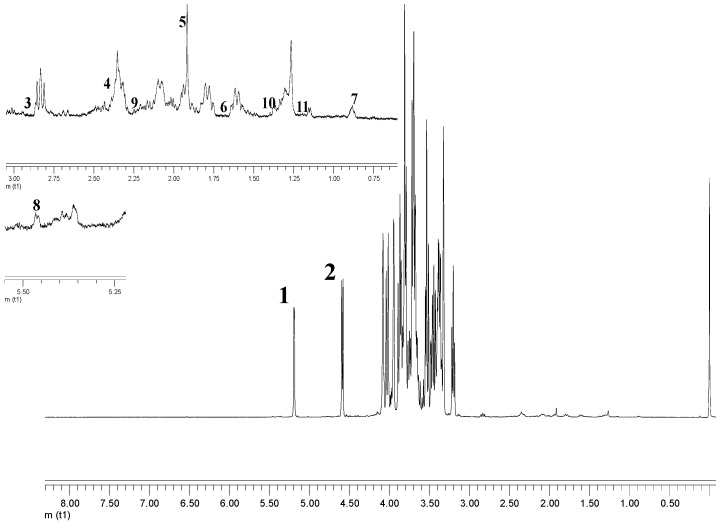
The representative ^1^H-NMR spectra of Grade 1 Ajwa from al-Aliah farm. Identified signals include: 1, beta glucose; 2, alpha glucose; 3, asparagine; 4, pyruvic acid; 5, acetate; 6, arginine; 7, steroid; 8, sucrose; 9, proline; 10, alanine; 11, linoleic acid.

Numerous beneficial health and pharmacological effects of Ajwa dates have been reported [6,9,10,11]. However, there has not been any scientific documentation that focused on the correlation of metabolite content with the grades of Ajwa dates. In this report, the discrimination of five samples of Ajwa dates from different origins and different grades was examined using multivariate data analysis on their ^1^H-NMR fingerprint, and the identification of important metabolites responsible for their discrimination was suggested. A total of 35 metabolites present in the hydrophilic extracts of Ajwa dates were characterized by nuclear magnetic resonance (NMR) spectroscopy. The representative spectrum of 1A is shown in Figure 1. Visual inspection of the ^1^H-NMR spectra of the extracts showed the presence of amino acids, sugars and organic acids. The most prominent signals in the spectra were in the sugar region. The signals attributable to amino acids were also identified and assigned to arginine and proline. The high field signals at δ 0.79 and 0.89 were assigned to steroids. The spectral data of the identified metabolites were also compared with those available in the literature data, as well as the search of the Chenomx database for confirmation. In addition, the assignments of the compounds were further supported by the results of 2D J-resolved NMR spectrum (Supplementary Materials Figure S1). The assigned metabolites are presented as in Supplementary Materials Table S1.

On the basis of the spectral analysis, only the Ajwa from al-Aliah farms (1A and 2A) contained succinic acid and concomitant absence of alanine. In addition, valine and lactic acid also were not detected in 1A, while 1B demonstrated the lack of pyruvic acid. All of the dates from the al-Aliah farm contained fumaric acid, except for 3A. Samples from Group 2A lacked kaempferol, epicatechin and gallic acid. It is also interesting to note that ascorbic acid was not detected in both Grade 3 Ajwa samples. Besides that, maleic acid and quercetin derivatives were also not identified in the Grade 3 Ajwa from Uhud (samples of 3U). The difference in metabolite composition of the different Ajwa samples can be attributed to several factors, such as geographical origin, cultivation practice and soil condition. The physiological function of plants may be altered by environmental factors, such as drought, which generally results in starch depletion and the accumulation of sugars in the leaves in order to provide protection against stress damage [12]. The effects of environmental factors towards the metabolite profile of plants have been previously reported [13].

### 2.2. Discrimination of Ajwa Samples on Unsupervised PCA of the NMR Finger Print

**Figure 2:**
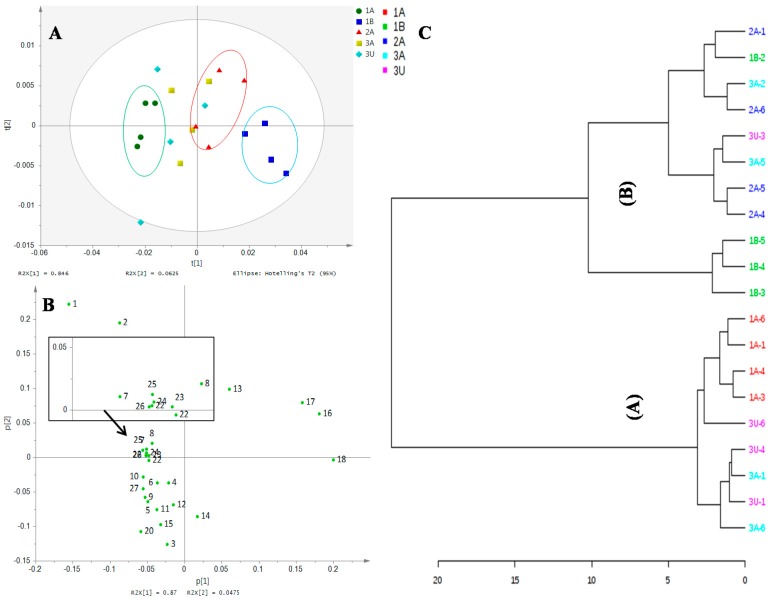
(**A**) The PCA score plot; (**B**) loading score plot; where; 1, beta glucose; 2, alpha glucose; 3, asparagine; 4, pyruvic acid; 5, acetate; 6, arginine; 7, steroid; 8, sucrose; 9, proline; 10, alanine; 11, linoleic acid; 12, glutamine; 13, fructose; 14, choline; 15, glutamic acid; 16, ascorbic acid; 17, glycine; 18, phenylalanine; 19, maleic acid; 20, succinic acid; 21, fumaric acid; 22, kaempferol; 23, quercetin; 24, gallic acid; 25, epicatechin; 26, cinnamic acid; 27, rhamnose in flavonoid; (**C**) hierarchical cluster analysis (HCA) dendrogram showing the relationship between Ajwa dates; 1A (Grade 1 from al-Aliah); 2A (Grade 2 from al-Aliah); 1B (Grade 1 from Bir Maashi); 3A (Grade 3 from al-Aliah) and 3U (Grade 3 from Uhud).

The ^1^H-NMR spectra of all Ajwa dates from different grades and different farms were subjected to preliminary evaluation of the discriminatory features of the selected dates. The unsupervised principal component analysis (PCA) analysis was conducted based on the datasets derived from the spectra to result in a scores plot, as shown in Figure 2A. High total variances were accounted for in the PC1 (84.6%) and PC2 (6.3%) of this plot. Clustering of groups is observed, although some of them may be more widely dispersed than the others and overlaps. The samples that belonged to 1A, 2A and 1B are quite well clustered and separated from one another. On the other hand, samples from the 3A and 3U groups are more dispersed and overlap with one another, as well as with the cluster of samples of 2A. Inspection of the PCA loading plot indicated that metabolites, including proline, glutamine, asparagine, arginine, glutamic acid, pyruvic acid, linoleic acid, fructose and glucose, contributed most to the separation of 1A samples (Figure 2B). In contrast, leucine, glycine, cinnamic acid and ascorbic acid are the metabolites that contribute to the discrimination of 1B samples.

The relationship between samples groups may be further clarified by the hierarchical cluster analysis (HCA) as shown in Figure 2C, The HCA dendrogram clearly revealed the formation of two major clusters designated as (A) and (B). The major cluster (A) comprised the samples from the 1A, 3A and 3U groups, which indicate the close relationship between these groups. In the major cluster (B), the samples from all other groups except 1A are represented. The absence of the sample from Group 1A in the cluster (B) and the sample from Group 1B in the cluster (A) indicates the distant relationship between the two groups. In the major cluster (B), two sub-clusters were further formed, one being mainly represented by 1B samples and the other by 2A samples. Due to the presence of multiple sources of variation that may affect the sample metabolite profile (grades and origin), distinct clustering between samples has become obscure, and overlapping between clusters becomes more apparent.

### 2.3. Differentiation of Ajwa Grades from al-Aliah Farm

**Figure 3:**
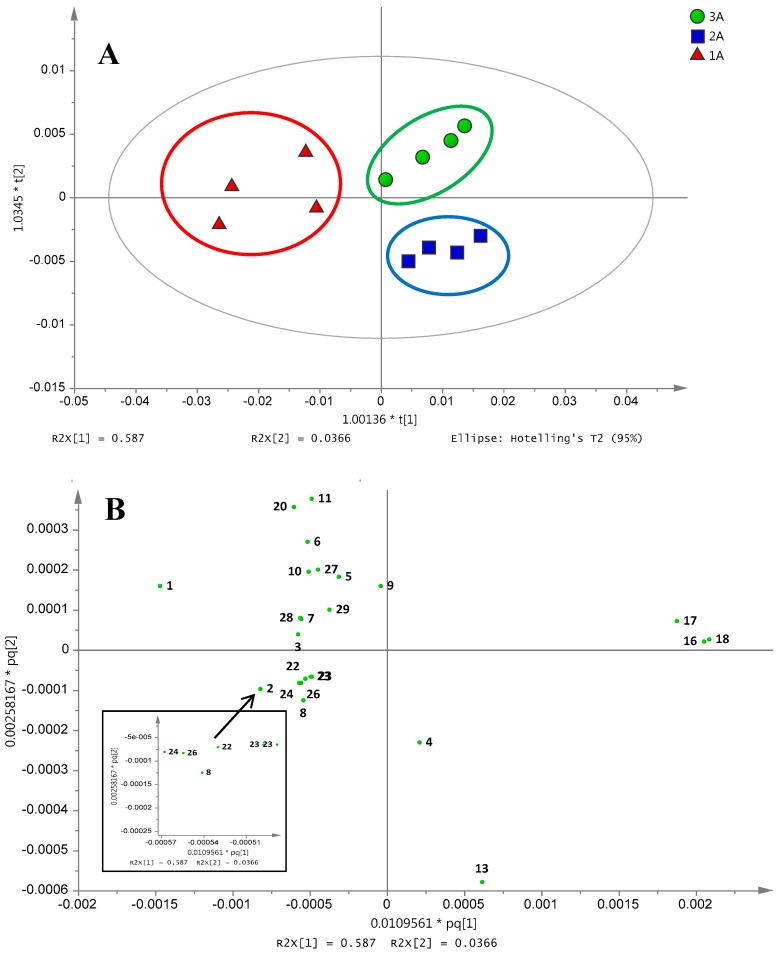
(**A**) The OPLS-DA score plot; (**B**) loading score plot of different grades of Ajwa dates from al-Aliah farm; 1A (Grade 1); 2A (Grade 2) and 3A (Grade 3); where; 1, beta glucose; 2, alpha glucose; 3, asparagine; 4, pyruvic acid; 5, acetate; 6, lactic acid; 7, steroid; 8, sucrose; 9, proline; 11, linoleic acid; 13, fructose; 16, ascorbic acid; 17, glycine; 18, phenylalanine; 20, succinic acid; 22, kaempferol; 23, quercetin; 24, gallic acid; 26, cinnamic acid; 27, rhamnose in flavonoid; 28, leucine; 29, valine.

The orthogonal partial least square discriminant analysis (OPLS-DA) was conducted to further understand the metabolic factors involved in the classification of three Ajwa samples from the same origin (al-Aliah) into three grades. Three clusters are distinctly observed, where 1A is separated from 2A and 3A by the first component (t[1]), while 2A is distinguished from 3A by the second component (t[2]; Figure 3A). The resulting OPLS-DA model appeared to attain goodness of fit with an R2Y cumulative value of 0.99 for two components and goodness of prediction at a Q2 value of 0.63. The sum of predictive and orthogonal variation (R2Xcum) of 0.95 was obtained. The significance of the model was also assessed using the *p*-value (*p* < 0.05) of CV-ANOVA. The *p*-value of 0.01 was acquired indicating that the obtained results were statistically significant. Based on the loading score plot of the respective OPLS-DA model (Figure 3B), the metabolites that contributed toward the separation of 1A from the other two Ajwa grades were succinic acid, linoleic acid, lactic acid, acetate, beta glucose, acetate, proline, valine, asparagine, kaempferol, cinnamic acid, gallic acid, quercetin and sucrose. Meanwhile, glycine, phenylalanine and ascorbic acid were positioned on the right side of the top quadrant, which corresponded to the position of 3A in the score plot. The one by one analysis was performed to assess the variation between 1A samples from each grade, 2A and also 3A Ajwa dates. The 1A was separated from 2A and 3A samples by the lower abundance of glycine, phenylalanine, ascorbic acid and fructose (Supplementary Materials Figure S2). These results are consistent with the previously-reported analysis on differentiation of date samples using multivariate data analysis based on inductively-coupled plasma data [14].

The robustness of the OPLS-DA model for classification of the sample was evaluated by creating the “probability of class membership” list. The list comprises an artificial Y-matrix of dummy variables, with the number of classes preferably in the range of 2–4 classes. The accuracy of prediction for the sample was determined by the “probability of class membership”, where values greater than 0.65 indicate the correct sample prediction while values between 0.35 and 0.65 were considered borderline [15]. The probability of the class membership list derived from the cross-validation set of the OPLS-DA model of the three grades of al-Aliah samples is presented in Table 1.

All of the samples labelled 1A and 2A were found to be correctly classified in the respective groups. However, for the samples labelled as 3A, two of the datasets were classified as borderline cases. The results from the construction of the probability of membership list suggested that 3A test samples were closely related to the 1A group, although they are distantly graded, which was in agreement with the results shown in HCA (Figure 2C). The grading of dates was commonly conducted by date growers based on the fruits size and absence of visual defects [16]. The sorting was done manually by workers; hence, some processing issues may occur resulting in the potential misclassification [17].

The misclassification table was used to confirm the discrimination between different grades of Ajwa dates collected from al-Aliah farm. This analysis was done by calculating the number of samples that were correctly predicted. The results from misclassification table construction showed that all samples have been classified with 100% accuracy (Supplementary Materials Table S2).

### 2.4. Differentiation of Ajwa Based on Farm Origins

**Figure 4:**
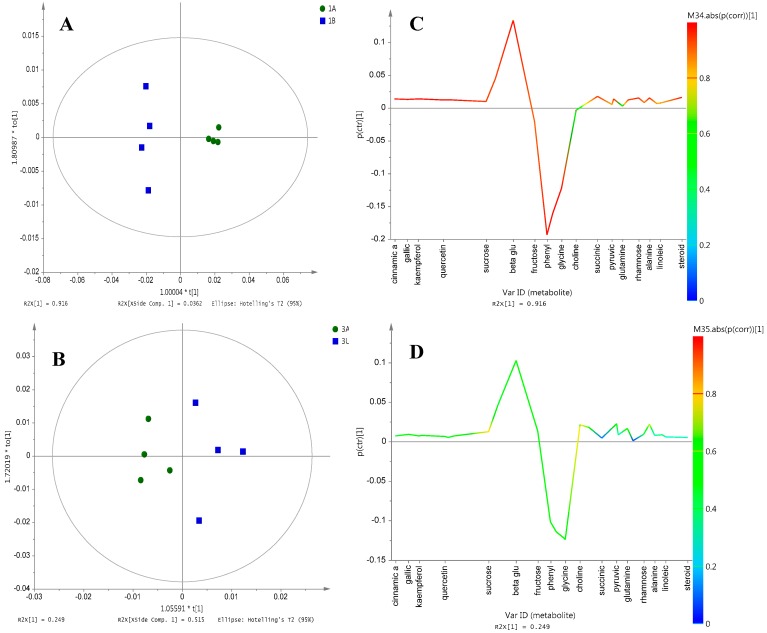
(**A**,**B**) OPLS-DA score plot; (**C**,**D**) OPLS-DA S-line plot for Grade 1 Ajwa from different cultivation farms; 1A (Grade 1 from al-Aliah); 1B (Grade 1 from Bir Maashi); 3A (Grade 3 from al-Aliah) and 3U (grade 3 from Uhud).

OPLS-DA was conducted, which allows the generation of the consequent S-line plot, hence enabling the detection of chemical markers for the resulting separation. The advantage of OPLS-DA over the PLS-DA method was that only one component was employed as the predictor for class [18], which can be achieved by the inclusion of the two classes of the sample groups. The OPLS-DA model for differentiation of Grade 1 Ajwa from different farms, Samples 1A and 1B, showed a distinct cluster separation along the first PC (Figure 4A). The model developed for these sample groups gave the sum of predictive and orthogonal variation (R2Xcum) of 0.96. The total sum of variation in Y explained by the model (R2Y) was 0.99, and the goodness of prediction Q2 was 0.95. A similar model developed on Grade 3 samples, represented by 3A and 3U, also exhibits a similar trend (Figure 4B).

The contribution of the metabolites responsible for the separation is visualized through the OPLS-DA S-line plot. This plot was specially made for NMR spectroscopy data and can be used to assist model interpretation of the OPLS-DA model with two classes. The extent of contribution is represented by colors (from blue to red) of the correlation column, with red being the highest and blue the lowest [19]. Cinnamic acid, gallic acid and quercetin were among the compounds that strongly influenced the discrimination of 1A from 1B (Figure 4C). The results as shown in Figure 4C,D were consistent with the observations of the loading column plot (Supplementary Materials Figure S3), which suggested that the compounds illustrated in Figure 4C displayed high reliability as chemical markers since their respective standard deviations did not cross the X-axis of the column plot [20]. In contrast, all metabolites in Grade 3 samples (3A and 3U) did not show a strong correlation with their contribution to separation, and this is reflected by the weak separation between clusters in the scores plot (Figure 4D). The weak separation between these Grade 3 samples may be rationalized by the close proximity of the al-Aliah and Uhud farms. The distinct separation and metabolite profiles between the al-Aliah and Bir Maashi could be explained by the large distance between the two locations. A different study has also suggested that environmental and agronomic conditions can affect the metabolite profile of plants [21].

### 2.5. NO Inhibitory Activity of the Different Grades of Ajwa Dates

**Figure 5:**
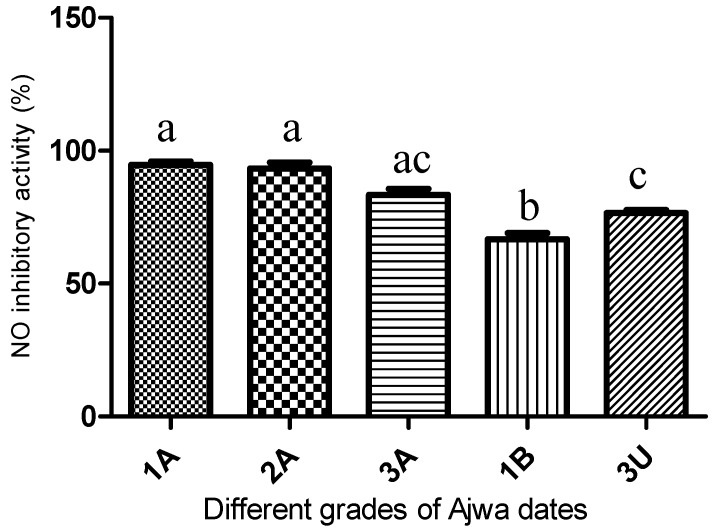
The percentage inhibition of NO inhibitory activity obtained from different grades of Ajwa with Samples 1A (Grade 1 from al-Aliah); 1B (Grade 1 from Bir Maashi); 3A (Grade 3 from al-Aliah) and 3U (Grade 3 from Uhud). The presented values are the mean of four replicates ± SD. Each different letter (a, b and c) above the column indicates that the results are statistically significant differences (*p* < 0.05; *n* = 6) and the letter “ac” is not significant different from “a” and “c”.

Nitric oxide (NO) inhibitory activity was measured in order to correlate the potential health benefits with the grades and origin of the samples, and the results are presented in Figure 5. All of the al-Aliah samples (1A, 2A and 3A) exhibited higher activity than the samples from other origins, with Sample 1A being the highest (95%) and 1B being the lowest (67%) (Supplementary Materials Table S3). Curcumin, which served as the positive control, was found to inhibit the production of NO by 97% at 0.1 mg/mL. All of the tested Ajwa samples exhibited high cell viability even at high sample concentration; thus, the observed inhibition was not due to the cytotoxicity effect (Supplementary Materials Table S3). From the overall results, it appears that farm origin, which may be translated into environmental factors and agricultural practices, contribute to a greater impact on the NO inhibitory activity than the difference in grades.

### 2.6. Correlation between Metabolite Profiles and NO Inhibitory Activity

**Figure 6:**
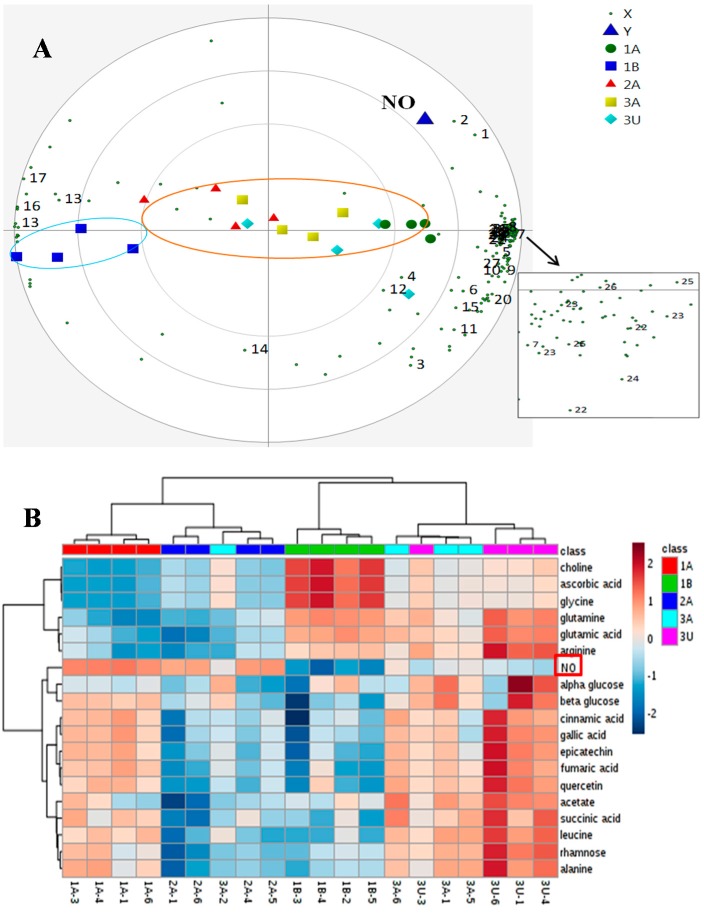
(**A**) PLS biplot where; 1, beta glucose; 2, alpha glucose; 5, acetate; 6, arginine; 9, proline; 10, alanine; 12, glutamine; 13, fructose; 14, choline; 16, ascorbic acid; 17, glycine; 19, maleic acid; 20, succinic acid; 21, fumaric acid; 22, kaempferol; 23, quercetin; 24, gallic acid; 25, epicatechin; 26, cinnamic acid; 27, rhamnose in flavonoid; 28, leucine; (**B**,**C**) the heatmap visualization of metabolite variation and NO inhibition of different Ajwa dates; 1A (Grade 1 from al-Aliah); 1B (Grade 1 from Bir Maashi); 3A (Grade 3 from al-Aliah) and 3U (Grade 3 from Uhud).

PLS analysis was performed to establish the correlation between metabolites and NO inhibitory activity. The observed NO inhibitory activity was assigned as the Y variable and chemical shifts as the X variables. The model developed from the spectral and biological data gave the total sum of variation in Y explained by the model (R2Y) at 0.83 and goodness of prediction Q2 at 0.61. The PLS biplot shows good separation of the samples into two groups with the samples from al-Aliah and Uhud farms clustered in the right quadrant, while the samples from Bir Maashi farms were all located in the left quadrant (Figure 6A). Metabolites that contribute to the high NO activity shown by 1A are also presented in the same plot. This suggests the role of amino acids (glutamine, arginine, leucine, proline), fumaric acid, maleic acid, succinic acid, cinnamic acid, sugars (alpha glucose and beta glucose) and phenolic compounds (epicatechin, rhamnose in flavonoid, quercetin derivative, kaempferol and gallic acid) as NO inhibitory contributors (Figure 6A).

The information regarding the correlation of metabolites and NO inhibitory activity was extended by constructing a heatmap using Ward’s method highlighting the metabolites obtained from previous PLS analysis. In the analysis, the ratio of relative concentration for the given metabolites and NO inhibitory activity was computed between the five different Ajwa dates (represented by different colors). In the heat map developed from these data (Figure 6B), the significance of metabolites and NO inhibition is displayed in red for high and blue for low. The shade of each color is consistent with the degree of variation in Ajwa dates [22]. In terms of NO inhibitory activity, the intensity of red color for 1A and 2A was almost the same, while that of 3A was spread across the dendrogram (horizontal axis) with the same color intensity as those for 3U. This was consistent with the NO inhibition of 3A, which was not significantly different from those of the 1A, 2A and also 3U samples (Figure 5). With regards to the metabolite profiles, the heat map visualization demonstrated that 1A samples contained a high prevalence of phenolic compounds, as well as beta glucose. The dendrogram (vertical axis) displayed a closer relationship of NO with these metabolites, hence suggesting that they are contributing more towards the high NO inhibition of Ajwa dates. This observation was in agreement with the findings obtained in PLS analysis (Figure 6A). The role of the metabolites detected in this study has been described in numerous studies [23,24,25]. Earlier studies have reported that phenolic compounds inhibit the production of NO in LPS-stimulated RAW 264.7 cells [26,27,28]. It is most likely that the greater NO inhibitor capacity shown by 1A was mainly influenced by the higher amount of phenolic compounds.

## 3. Materials and Methods

### 3.1. Materials

Deuterated methanol-*d*_4_ (CD_3_OD, 99.8%), non-deuterated KH_2_PO_4_, sodium deuterium oxide (NaOD), 3-(trimethylsilyl)propionic acid-*d*_4_ sodium salt (TSP) and deuterium oxide (D_2_O, 99.9%) were purchased from Merck (Darmstadt, Germany). Curcumin, lipopolysaccharide (LPS), phosphate-buffered saline (PBS) and recombinant murine interferon-gamma (IFN-γ) were obtained from Sigma-Aldrich Co. (St. Louis, MO, USA). The cell culture media, Dulbecco’s Modified Eagle’s Medium (DMEM) containing both HEPES and l-glutamine with phenol red and that without phenol red, penicillin-streptomycin antibiotic solutions, fetal bovine serum (FBS), 3-(4,5-dimethylthiazol-2-yl)-2,5-diphenyltetrazolium bromide (MTT) and triple Express enzyme were acquired from Gibco/BRL Life Technologies Inc. (Eggenstein, Germany).

### 3.2. Fruit Materials

Three different grades of Ajwa fruits were obtained from different farms in the District of Madinah, Saudi Arabia (SA). The sample assigned as 1A was obtained from al-Aliah farm, which is located approximately 3–4 km due south from the city center, while the sample assigned as 1B was the produce from the farm in Bir Maashi District, which is located about 60 km away from the city. Both of these samples are classified as Grade 1 products. Grade 2 Ajwa dates from al-Aliah farm (2A) were also included in the current study. The Grade 3 Ajwa were represented by the sample assigned as 3A (from al-Aliah District) and 3U (from Uhud, located about 3 km due north from the city center). The altitudes of all of the farms are approximately 600 m above sea level. The soil of the date farms is kept moist, and each palm tree is irrigated through small channels (20 cm deep × 20 cm wide) from the water supply, which is extracted from the ground. To ensure the healthy growth of trees, fertilization is conducted every four months using chemical NPK (3:2:5 ratios). All date samples were collected at the tamar stage from the respective farms and also received similar treatment.

### 3.3. Sample Preparation

Twenty fruits from each of the five Ajwa samples were cleaned from impurities. Each sample group is then divided into four parts (five fruits each) representing four sample replicates. The fruits were pitted, cut into small pieces and stored at −80 °C before being freeze-dried. The dried date fruits (1.0 g) were extracted by stirring with 30 mL of absolute methanol at room temperature for 24 h. Following this, the extract was filtered using Whatman filter paper No. 1 in a Buchner funnel, and the filtrate was concentrated to dryness under vacuum with a rotary evaporator. All samples were then stored in the dark at 4 °C until further analysis [29,30]. NMR data were obtained for all four replicates.

### 3.4. NMR Measurement

The 1D ^1^H-NMR spectra were taken at 500 MHz on a Varian INOVA NMR spectrometer (Varian Inc., Palo Alto, CA, USA) at a running frequency of 499.887 MHz, and the temperature was maintained at 26 °C. The acquisition time for each ^1^H-NMR spectrum was 3.53 min to accomplish 64 scans for the spectral width of 20 ppm. Suppression of the large water resonance was achieved by applying a presaturation pulse sequence to the ^1^H-NMR data acquisition. For each sample, a relaxation delay was set at 1.0 s. Ten milligrams of the dried extracts were transferred into 2-mL Eppendorf tubes containing 0.375 mL of CD_3_OD. Then, 0.375 mL of KH_2_PO_4_ buffer in D_2_O (pH 6.0) containing 0.1% TSP were added as the buffering agent. The tube was vortexed and then ultra-sonicated for 10 min at room temperature. The solution was then centrifuged at 13,000 rpm for 10 min. The resulting supernatant (0.6 mL) was transferred into a 5-mL NMR tube for ^1^H-NMR analysis [31]. The chemical shifts of all datasets were referenced to the internal standard, trimethylsilylpropionic acid-*d*_4_ sodium salt (TSP).

### 3.5. Nitric Oxide Inhibitory Activity

The RAW 264.7 murine macrophage cells, obtained from the American Type Culture Collection (ATCC, Rockville, MD, USA), were grown in plastic culture flasks in Dulbecco’s Modified Eagle’s Medium (DMEM) with phenol red, which also contained HEPES, l-glutamine supplemented with 10% fetal bovine serum (FBS) and 1% antibiotic solution (Gibco/BRL). The culture was maintained under 5% CO_2_ at 37 °C. After 3–4 days, the cells were collected from the flask by using TrypLE™ Express enzyme, followed by centrifugation at 1000 rpm and 4 °C for 10 min. The supernatant was discarded and the cells were re-suspended in fresh DMEM (without phenol red) containing HEPES and l-glutamine and other similar supplements. The cells were then counted, and the viability was determined by the typical trypan blue cell counting technique. The concentration of the cell was adjusted to 1 × 10^6^ cells/mL in the same medium. The cells were then cultured in the previously-mentioned media (50 µL) with the addition of the triggering agents (200 U/mL of recombinant murine IFN-γ and 10 µg/mL lipopolysaccharide), seeded into the wells of 96-well culture plates. A serial dilution of the fruit extract was conducted in the medium containing DMSO. Fifty microliters of the prepared extracts (10.0, 5.0, 2.50, 1.25, 0.63, 0.31 and 0.156 mg/mL) were loaded into the wells of cell plates to yield the final concentration of 0.4% DMSO per well. The experiment was performed in six replicates, and the cells were incubated for 17 h at 37 °C, 5% CO_2_ in a humidified incubator. The controls used in this experiment were curcumin as the positive control, blank medium, while the other two controls are the cells cultured in medium only and that cultured in the medium containing triggering agents with 0.4% DMSO [32].

#### 3.5.1. Measurement of Nitrite

The stable nitric oxide conversion product, nitrite (NO_2_^−^), was measured using the Griess reagent (1% sulfanilamide, 0.1% *N*-(1-naphthyl)-ethylenediamine dihydrochloride and 2.5% H_3_PO_4_). After 17 h of incubation, 50-µL aliquots were removed from the supernatant of the cultured cells and incubated with an equal volume of the Griess reagent at room temperature for 15 min. The absorbance was taken at 550 nm using a (SpectraMax PLUS) UV/Vis microplate reader. The percentage of inhibition was calculated for all samples. Fresh culture medium was used as a blank in every experiment [32].

#### 3.5.2. Cell Viability

The cytotoxicity assay was performed to validate the NO inhibition activity by evaluating the viability of the treated cells. After the removal of the media, 100 µL of complete DMEM were loaded into the wells. Following this, 20 µL of a 5-mg/mL solution of MTT in phosphate-buffered saline (PBS) 7.2 were added. The cells were incubated at 37 °C in 5% CO_2_ for 4 h. The absorbance was read at 570 nm. The absorbance of formazan salt in the control (untreated cells) was taken as 100% viability, where the percentages of dead cells were calculated in comparison to the control group [32].

#### 3.5.3. Data Analysis

The ^1^H-NMR spectra were phased, base-lined corrected and binned using Chenomx software (v. 5.1, Edmonton, AB, Canada). The spectral region of 0.50–10.00 ppm was bucketed into sections of 0.04 ppm each. The binned ^1^H-NMR data were then subjected to multivariate data analysis performed with SIMCA-P+ Version 13.0 (Umetrics AB, Umeå, Sweden) software. To examine the metabolite multi-dimensional dataset, both the unsupervised and supervised approach were applied [33,34,35,36,37]. The identification of compounds was achieved using two-dimensional 2D J-resolved spectroscopy [38]. Other than that, additional analysis was carried out by generating a dendrogram and heatmap using the MetaboAnalyst data approach, a web-based platform for metabolomics data analysis [38].

### 3.6. Statistical Analysis

GraphPad PRISM^TM^ software was used (Graph Pad Software, Inc., San Diego, CA, USA). Results are expressed as the mean of four replicates ± SD. Statistical significance between groups was analyzed by one-way ANOVA. The significance level was set at *p* < 0.05.

## 4. Conclusions

In this study, the discrimination of Ajwa date samples from different grades and origins available on the market has been achieved using multivariate analysis of the ^1^H-NMR spectra. Among the reliable chemical markers in the separation of 1A from the other al-Aliah dates were phenolic compounds, ascorbic acid and phenylalanine. It is worthy to note that the same compounds also strongly influenced the discrimination of Ajwa dates of al-Aliah from Bir Maashi origins. The results revealed that Ajwa dates of different grades and origins possessed different metabolite profiles and compositions and, on the basis of these, exhibit varied NO inhibitory activity. The study also found that Ajwa dates from al-Aliah farms are more active as an NO inhibitor as compared to those from other origins, with Sample 1A being the highest and that produced in Bir Maashi being the least. This finding supports the Prophetic claim that Ajwa dates from al-Aliah are better among all of the others. The Grade 1 samples were stronger than Grade 2, followed by Grade 3, although the differences are not highly significant. The samples from Uhud were stronger than those from Bir Maashi, but closer to the al-Aliah samples, which may be associated with their close geographical location and possibly sharing the same underground hydrological system. This study also demonstrated that farm origin affects more the NO inhibitory activity of the date fruits as opposed to the grades. The results presented in this report offers important information regarding the quality of Ajwa dates to consumers.

## Figures and Tables

**Table 1:** Classification of membership list for the discriminant analysis model.

Origin	Predicted Y Value for Dummy Variables		
	Aliah 1	Aliah 2	Aliah 3
Aliah 1	**1.08**	−0.17	0.09
Aliah 1	**0.74**	−0.25	0.52
Aliah 1	**1.12**	0.12	−0.23
Aliah 1	**0.65**	0.24	0.11
Aliah 2	−0.19	**0.93**	0.26
Aliah 2	0.15	**0.94**	−0.09
Aliah 2	−0.08	**1.00**	0.08
Aliah 2	0.06	**0.89**	0.05
Aliah 3	0.32	0.19	**0.48**
Aliah 3	0.15	0.11	**0.74**
Aliah 3	0.03	0.04	**0.93**
Aliah 3	−0.03	−0.04	**1.07**

## Supplementary Materials

Supplementary materials can be accessed at: http://www.mdpi.com/1420-3049/21/11/1423/s1.
