# Supplementary material for: Discrimination and Nitric Oxide Inhibitory Activity Correlation of Ajwa Dates from Different Grades and Origin
Source: Molecules. 2016 Oct 28;21(11):1423. doi: 10.3390/molecules21111423 (PMC6274092; doi:10.3390/molecules21111423)
Supplement: Supplementary file 1 [file molecules-21-01423-s001.pdf]

Supplementary Materials: Discrimination and Nitric Oxide Inhibitory Activity Correlation of Ajwa Dates from Different Grades and Origin

Nur Ashikin Abdul-Hamid, Ahmed Mediani, M. Maulidiani, Faridah Abas, Intan Safinar Ismail, Khozirah Shaari and Nordin H. Lajis

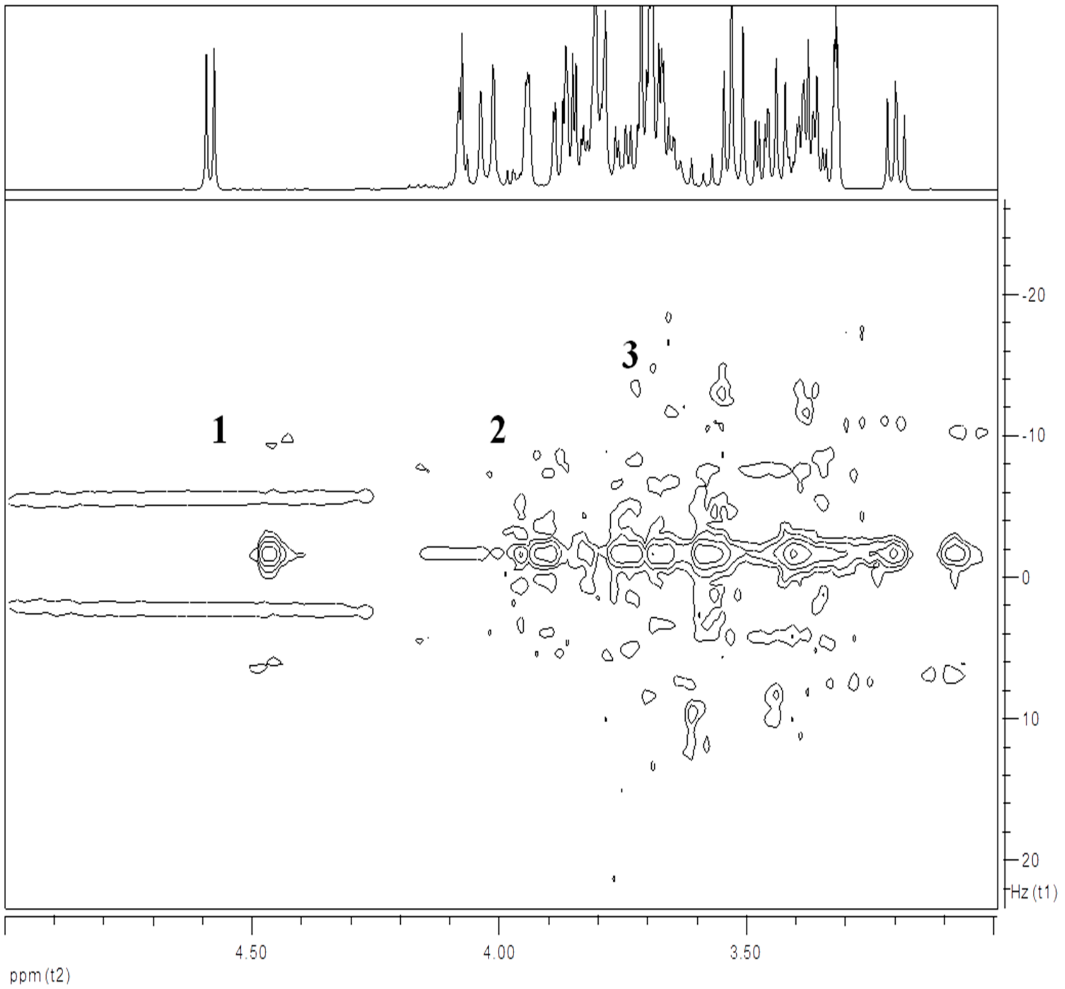

Figure S1. The 2D J-resolved spectra of grade 1 al-Aliah expanded in the sugar region; 1, beta glucose; 2, fructose; 3, ascorbic acid.

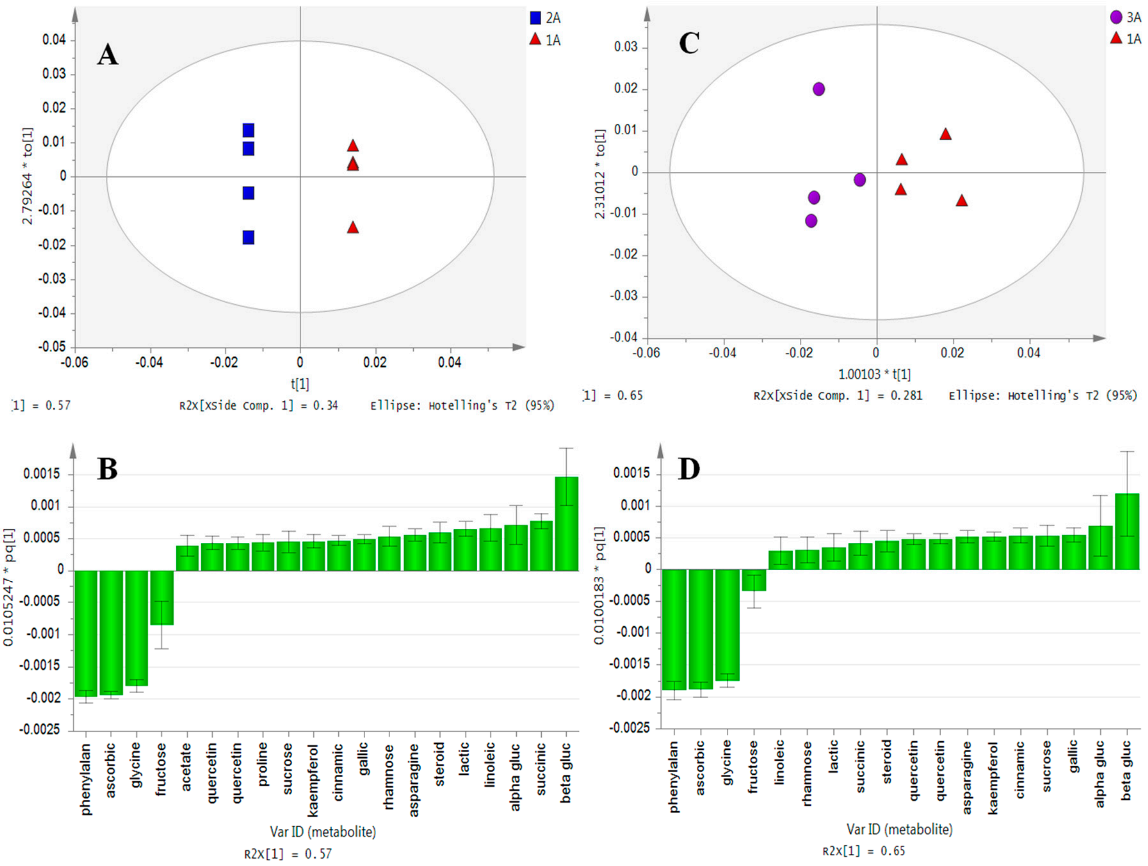

Figure S2. OPLS-DA scores plot (**A**) 1A versus 2A and (**B**) corresponding loading column plot (**C**) 1A versus 3A and its (**D**) corresponding loading column plot; 1A (grade 1 from al-Aliah), 2A (grade 2 from al-Aliah) and 3A (grade 3 from al-Aliah).

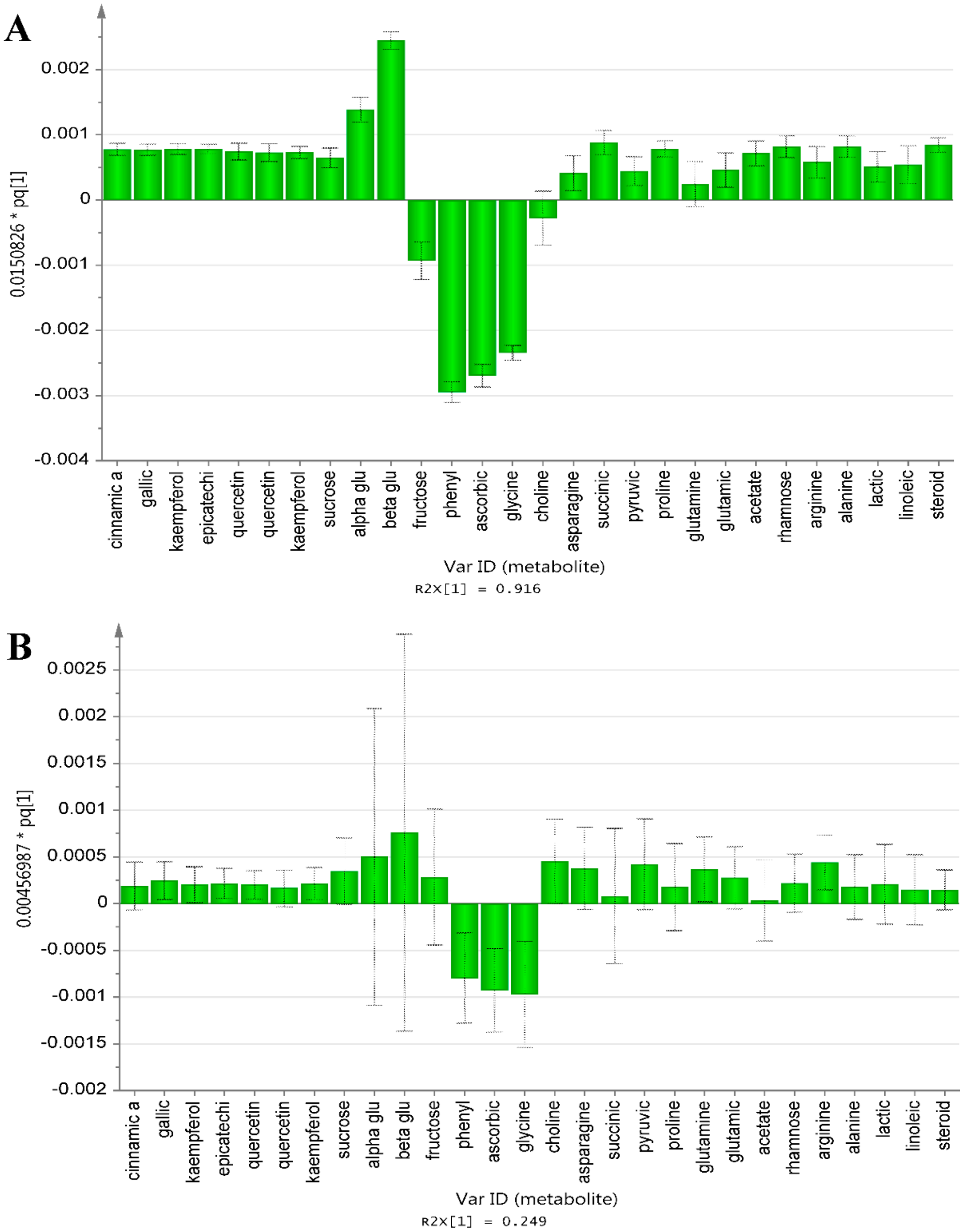

Figure S3. OPLS-DA loading column plot (**A**) 1A versus 1B (**B**) 3A versus 3U. 1A (grade 1 from al-Aliah), 1B (grade 1 from Bir Maashi), 3A (grade 3 from al-Aliah) and 3U (grade 3 from Uhud).

Table S1. Assignments of 1D and 2D NMR spectral signals attained from different grades of Ajwa dates where; (s: singlet, d: doublet and m: multiplet) and identification of the respective compounds.

Compounds	Chemical Shift
Steroid	0.79 (s), 0.89 (s)
Alanine	1.46 (d, 7.5)
Arginine	1.60–1.75 (m)
Asparagine	2.73–2.78 (m), 2.77–2.90 (m)
Proline	1.99–2.09 (m)
Valine	2.28 (m)
Phenylalanine	7.32–7.33 (m), 7.38 (m)
Betaine	3.27 (s), 3.85 (s)
Inosine	4.22 (m), 4.38 (m), 4.69 (m)
Choline	3.30 (s)
Glutamine	2.07–2.17 (m), 2.00–2.10 (m)
Glycine	3.53 (s), 3.57 (s)
Isoleucine	0.89 (t, 7.38 Hz)
Leucine	0.94 (t, 6.06 Hz)
Acetate	1.90 (s)
Lactic acid	1.31 (d, 6.5 Hz)
Linoleic acid	1.23–1.38 (m)
Glutamic acid	1.92–2.05 (m)
Pyruvic acid	2.38 (s)
Succinic acid	2.63 (s), 2.66 (s)
Ascorbic acid	3.72–3.76 (m), 3.70–3.73 (m)
Maleic acid	6.13 (s), 6.39 (s)
Fumaric acid	6.62 (s)
Cinnamic acid	7.40–7.44 (m)
Rhamnose in flavonoid	1.38 (s), 1.52 (s), 1.70 (s)
Quercetin derivatives	6.19 (d, 2.0 Hz), 6.28 (d, 1.8 Hz), 6.33 (d, 1.5 Hz), 6.99 (d, 8.6 Hz)
Kaempferol	6.17 (d, 2.0 Hz), 6.24 (d, 2.0 Hz), 6.31 (d, 2.0 Hz), 6.72 (d, 8.6 Hz), 6.91 (d,8.0 Hz)
Epicatechin	6.89–6.91 (m), 6.98–7.01 (m)
Gallic acid	7.01 (s)
Beta Glucose	4.47–4.49 (d, 8.0 Hz), 4.6 (d, 8.0 Hz)
Alpha Glucose	5.2 (d, 8.0 Hz)
Sucrose	5.40 (d,8.0 Hz), 4.19 (d, 8.7 Hz)
Fructose	4.12 (d, 8.6 Hz)
Xylose	4.57 (d, 8.1 Hz)

Table S2. The misclassification table of classes in OPLS-DA.

Ajwa from al-Aliah Farm	Members	Correct	3A	2A	1A
3A	4	100%	4	0	0
2A	4	100%	0	4	0
1A	4	100%	0	0	4
Total	12	100%	4	4	4

Table S3. Nitric oxide (NO) and percentage cell viability (MTT assay) obtained from different grades of Ajwa dates 1A (grade 1 from al-Aliah), 2A (grade 2 from al-Aliah),1B (grade 1 from Bir Maashi), 3A (grade 3 from al-Aliah) and 3U (grade 3 from Uhud).

**Different Grades of Ajwa Dates**		
**Samples**	**NO Inhibition (%)**	**Cells Viability %**
1A	94.83 ± 1.33 [a]	78.01
2A	93.77 ± 1.05 [a]	97.24
3A	81.63 ± 1.25 [ac]	91.08
1B	66.67 ± 2.34 [b]	95.36
3U	76.52 ± 1.21 [c]	97.76
